# Transoral Robotic Surgery in Head and Neck Oncology: A Scoping Review of Evidence, Technological Advancements, and Future Trajectories

**DOI:** 10.1002/jso.70353

**Published:** 2026-08-16

**Authors:** Anna Luíza Damaceno Araújo, Karthik N. Rao, Juan P. Rodrigo, Vincent Vander Poorten, Orlando Guntinas‐Lichius, Alfio Ferlito, Luiz Paulo Kowalski

**Affiliations:** ^1^ Head and Neck Surgery Department and LIM 28 University of São Paulo Medical School São Paulo Brazil; ^2^ Hospital Israelita Albert Einstein São Paulo Brazil; ^3^ Department of Head and Neck Oncology Sri Shankara Cancer Foundation Bangalore India; ^4^ Department of Otolaryngology, Hospital Universitario Central de Asturias, ISPA, IUOPA, CIBERONC University of Oviedo Oviedo Spain; ^5^ Department of Oncology, Section Head and Neck Oncology KU Leuven Leuven Belgium; ^6^ Otorhinolaryngology—Head and Neck Surgery, Leuven Cancer Institute University Hospitals Leuven Leuven Belgium; ^7^ Department of Otorhinolaryngology Jena University Hospital Jena Germany; ^8^ International Head and Neck Scientific Group Padua Italy; ^9^ Department of Head and Neck Surgery and Otorhinolaryngology A.C. Camargo Cancer Center São Paulo State of São Paulo Brazil

**Keywords:** bleeding, HPV positive, oropharyngeal cancer, quality‐of‐life, robotic, survival

## Abstract

Robotic surgery has transformed head and neck cancer (HNC) treatment. This scoping review synthesizes evidence on transoral robotic surgery (TORS), focusing on applications, limitations, and future directions. Following PRISMA‐ScR guidelines, this review used the PCC framework (Population: HNC patients; Concept: technological advancements; Context: any clinical setting). A systematic search of five databases and gray literature was conducted. Two independent reviewers screened records and extracted data from clinical trials and reviews. From 5411 records, 21 studies were included. Evidence confirms TORS provides excellent local disease control and functional benefits (e.g., shorter hospitalization, improved swallowing) over open surgery, albeit with a distinct complication profile (e.g., hemorrhage). A significant finding is the scarcity of high‐quality randomized trials comparing TORS to radiotherapy or open surgery. Technologically, evolution from multiport to single‐port and flexible systems has improved access, though limitations like absent haptic feedback persist. Emerging trends focus on AI integration for precision and developing smaller instruments. Robotic surgery is a viable, evolving approach for HNC with demonstrable functional advantages. Its future depends on overcoming technological limits, establishing cost‐effectiveness, and validating efficacy through robust comparative studies. AI integration is a promising frontier for enhancing surgical precision and personalization.

## 1 Introduction

The management paradigm for head and neck cancer (HNC) has progressively shifted toward minimally invasive, transoral approaches to reduce treatment‐related morbidity while preserving oncologic efficacy. TORS represents a pivotal advancement in this domain, enabling precise resection of select early‐stage, residual, and recurrent tumors. Its integration with neck dissection and reconstructive techniques underscores its role in comprehensive surgical management [1]. Compared to open surgery, TORS demonstrates a superior safety profile while achieving comparable local disease control, with a distinct complication spectrum, primarily postoperative hemorrhage and trismus, that contrasts with the long‐term toxicities of radiotherapy [2]. Despite established clinical applications, a systematic synthesis of the evolving evidence, technological integration, and future trajectory of TORS is lacking.

This scoping review (ScR) therefore aims to critically map the current landscape and project future developments in TORS for HNC. It will synthesize recent clinical advances, evaluate technological innovations, address existing limitations, and delineate research priorities for the coming decade. The guiding question is: “What are the contemporary advances and future directions of TORS in head and neck oncology?”

## 2 Methods

### 2.1 Protocol

This ScR follows the Preferred Reporting Items for Systematic reviews and Meta‐Analyses extension for Scoping Reviews (PRISMA‐ScR) Checklist [3]. This protocol was registered on Open Science Framework (OSF), available online at https://osf.io/rduvj.

### 2.2 Eligibility Criteria

The ScR question and the eligibility criteria were defined according to the PCC framework, in which: Population (P) referred to patients diagnosed with HNC, who underwent surgical treatment involving any robotic‐assisted technique; Concept (C) encompassed technological advancements and future directions of robotic surgery in head and neck oncology; and Context (C) included any clinical or research setting, including hospital‐based studies, multicenter projects, registries, or prospective technological development initiatives, without geographical restrictions.

Eligible studies included clinical trials, representing the highest level of current evidence, as well as literature reviews and systematic reviews (SRs). There were no restrictions on language or year of publication. Studies were excluded if they (i) involved robotic surgery exclusively for nononcological procedures, (ii) did not focus on robotic techniques, (iii) assessed quality of life (QOL), (iv) reported medical teams' experience or training through surveys, (v) clinical trials with incomplete recruitment, results pending or follow‐up ongoing, and cost‐effectiveness studies. Clinical trials will be assessed to retrieve the current evidence on TORS, while reviews will be evaluated to summarize advances, latest technological developments, current limitations, and ongoing projects as well as perspectives for the future.

### 2.3 Information Sources and Search Strategy

A comprehensive literature search was conducted on August 14, 2025 in the following electronic databases: Embase, The Regional Portal of the Virtual Health Library, PubMed/MEDLINE, Scopus, and Web of Science. The gray literature was explored through Google Scholar and ProQuest Dissertations & Theses. Additionally, the reference lists of included studies and relevant reviews were manually screened to identify additional eligible publications. The search strategy combined controlled vocabulary and free‐text keywords related to “robotic surgery” and “head and neck cancer.” The complete search strategies for all databases are provided in Appendix S1.

### 2.4 Selection of Sources of Evidence

All retrieved records were exported to Rayyan for deduplication. Two reviewers (A. L. D. A. and L. P. K.) independently screened titles and abstracts in the first selection phase. In the second phase, full‐text screening was performed applying the eligibility criteria. Any disagreements were resolved by discussion and, if necessary, by consulting a third reviewer (A. F.).

### 2.5 Data Charting Process and Data Items

Data extraction was performed by one reviewer (A. L. D. A.) and subsequently cross‐checked by a second reviewer (L. P. K.). For clinical trials, the following variables were extracted: author(s), year of publication, study type, patient number, and inclusion/exclusion criteria, interventions, outcomes analyzed, and results. A standardized data extraction form was developed to ensure consistency of data extraction.

### 2.6 Synthesis Methods

The findings are summarized using descriptive synthesis supported by tables to map the current evidence. Quantitative data are presented as frequencies or descriptive statistics where applicable. The synthesis organizes results chronologically and thematically as follows: (i) Current evidence on Efficacy and Safety, (ii) Comparison Between TORS and Transoral Laser Microsurgery (TLM), (iii) Applications, (iv) Technological Developments, (v) Current Limitations, and (vi) Emerging Technologies and AI Integration.

## 3 Results

### 3.1 Selection of Sources of Evidence

Among a total of 5411 records retrieved through the search strategy, 18 articles were deemed within the scope of this ScR. Additionally, 3 articles were identified through hand‐searching of the reference lists of the included studies, bringing the final total to 21. The study selection process is outlined in Figure 1, and the excluded articles, along with the respective reasons for exclusion, are detailed in Appendix S2.

**Figure 1:**
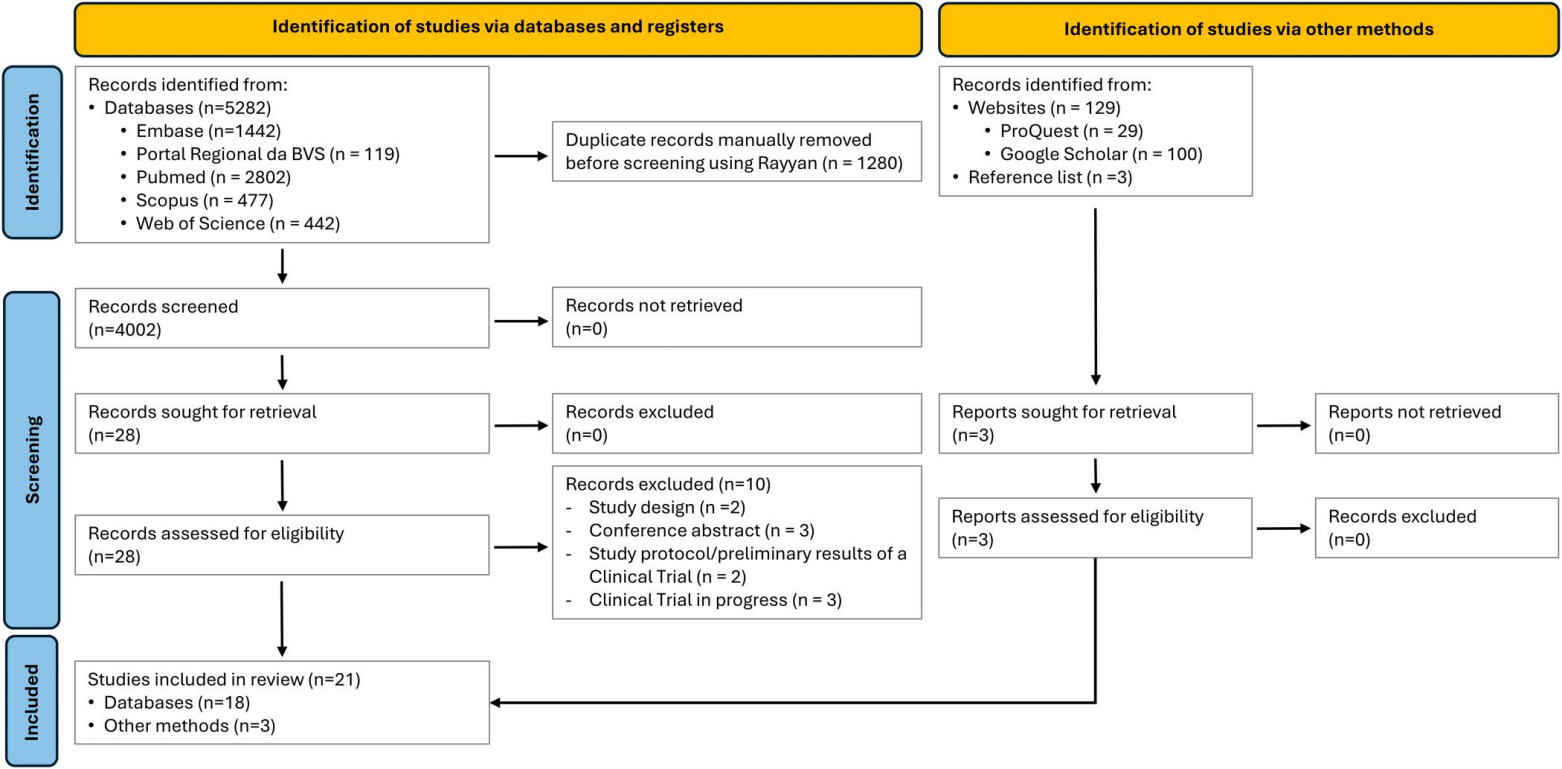
PRISMA 2020 flow diagram for new systematic reviews which included searches of databases, registers, and other sources. Adapted from Page et al. [4].

### 3.2 Characteristics of Sources of Evidence

A total of 15 clinical trials were retrieved from the database search [5, 6, 7, 8, 9, 10, 11, 12, 13, 14, 15, 16, 17, 18, 19], as well as 1 SR comparing TORS versus open surgery for HNC [20], 1 SR, and 4 literature reviews that highlighted the robotic advances in head and neck surgery, providing a comprehensive overview of the applications and evolution of robotic systems for TORS [21, 22, 23, 24]. Current limitations of TORS were extensively discussed across all included studies, while emerging technologies and future perspectives were reported in three studies that specifically discussed the relationship between robotics and Artificial Intelligence (AI) [25, 26, 27], with one of them focusing specifically on AI‐driven robotic surgery in the head and neck region [27].

### 3.3 Synthesis of Results

#### 3.3.1 Current Evidence on Efficacy and Safety

Relevant information on the included clinical trials and outcomes evaluated is summarized in Table 1. Current evidence on TORS shows clear functional benefits over open surgery, comparable oncological outcomes, and distinct complications. In general, studies show faster recovery, shorter hospitalization, and better swallowing function compared to open surgery. While TORS improves functional outcomes and minimizes invasiveness, its oncologic equivalence and cost‐effectiveness require further validation [21].

**Table 1:** Clinical trials included in the scoping review.

Author, year [citation]	Study type	*N*	Cancer type	Interventions	Outcomes analyzed	Results
Chan et al., 2019 [5]	Phase II single‐arm clinical trial (IDEAL Stage 2)	21	Nasopharyngeal, oropharyngeal, oral cavity, supraglottic glottic, pharyngeal, hypopharyngeal	TORS	Conversion rates	No conversions
					Mean docking time	7 min
					Mean operative (robotic) time	61.1 min
					Mean estimated blood loss	39.2 mL
					Mean LOS	10.4 days
					Surgical complications	Eight patients—unrelated to the TORS
Dabas et al., 2017 [6]	Single‐arm clinical trial	57	Oropharyngeal (HPV negative)	TORS	Robotic set‐up time	7.4 ± 2 min
					Mean operative (incision to closure) time	31.5 ± 5 min
					Mean blood loss	30 ± 9 mL
					Tracheostomy rate	6 patients (10.5%)—all successfully decannulated after surgery
					Feeding tube dependency	Four (7.1%) patients underwent percutaneous endoscopic gastrostomy, rest 53 patients had NGT placement. Two (3.5%) patients were dependent on long‐term NGT/PEG feeding.
					Time to oral feeds	Oral feeds within 2 weeks of procedure (mean 7.6 days), with the NGT removed within 2–3 weeks post‐op (mean 13.4 days).
					LOS	5.8 days (range, 4–9 days)
					Postoperative complications	Primary hemorrhage required active intervention in one patient. One patient developed aspiration pneumonitis which was managed conservatively.
					Recurrence rate	Two (4.2%) patients had locoregional recurrence
					DFS	43 (89.6%)
					OS	45 (93.8%)
de Almeida et al., 2024 [7]	Phase II single‐group nonrandomized controlled clinical trial (FIND)	22	Unknown primary squamous cell carcinoma of the neck	Diagnostic TORS vs. therapeutic TORS	Out‐of‐treatment‐volume failure	0%
					Locoregional control	100%
					Distant metastasis control	95%
					OS	100%
					Toxic effects (reported for entire cohort; not stratified by surgical intent)	*Grade 3 or 4 surgical toxic effects:* 2 patients (9%) (23%)
						*Grade 3 or 4 acute toxic effects:* 5 patients
						*Grade 3 or 4 late toxic effects:* 1 patient (5%)
					Swallowing outcomes (comparison by RT field; for MDADI by surgical intent, see the original study)	*MDADI:* associated with a smaller decline in swallowing scores during treatment (19.3 vs. 39.7; mean difference, −20.4; 95% CI, −34.1 to −6.1)
					VFSS swallow (comparison by RT field; not stratified by surgical intent)	*DIGEST:* associated with fewer patients with worsening DIGEST grade on videofluoroscopic swallow studies at 2 years (0% vs. 60%; difference, 60%; 95% CI, 30%–90%).
Ferris et al., 2022 [15]	Phase II randomized clinical trial	519	Oropharyngeal (HPV positive)	TORS + PORT	2‐year PFS	*A:* 96.9%; *B:* 94.9%; *C:* 96%; *D:* 90.7%
				Arm A (low risk, observation)		
				Arm B (intermediate risk, 50 Gy)	3‐year PFS	*A:* 96.9%; *B:* 94.9%; *C:* 93.4%; *D:* 90.7%
				Arm C (intermediate risk, 60 Gy)	2‐year OS	*A:* 100%; *B:* 99%; *C:* 98.1%; *D:* 96.3%
				Arm D (high risk, 66 Gy + cisplatin)	Toxicity (Grades 3–5)	*B:* 15%; *C:* 24%; *D:* 61%
					Quality of life/swallowing	Declined during treatment but returned to baseline in Arms A–C; remained lower in Arm D.
Haller et al., 2023 [8]	Single‐arm clinical trial (MC1273 or MC1675)	219	Oropharyngeal (HPV positive)	TORS + ND	Postoperative bleeding events	A total of 29 patients (13.2%) experienced a postoperative bleeding event. In total, 10 patients (4.6%) required operative intervention for bleeding.
					Postsurgical complications	*Emergency return:* 33 patients (15.1%)
						*Hospital readmission:* 10 patients (4.6%)
					Tracheostomy	Seven patients (3.2%). All were decannulated within 26 days.
					PEG tubes	Placed in two patients (0.9%)
					NGT	Placed intraoperatively in 96 patients (44.2%) for an average duration of 7.3 days
					LOS	Longer for patients with major bleeding (4.3 days vs. 2.9 days)
Holsinger et al., 2019 [9]	Two IDEAL Stage 2 multicenter clinical trials	47	Oropharyngeal	TORS	Feasibility	100% completion with SP system. Zero conversions to open/multiport robotics/laser
					Safety	No intraoperative complications; *Mean estimated blood loss:* 15 mL (no transfusions); *Margins:* 98% negative (1/40 positive)
					Adverse events	*Grades 1–2* (15 patients, 20 events): dysgeusia, dysphonia, minor bleeding (clinic‐managed)
						*Grades 3–4* (4 patients): 2 hemorrhages (post‐antithrombotic use); 1 *C. difficile enterocolitis*; 1 stroke (unrelated to device)
					Functional outcomes	*Diet:* 96% oral intake by 30 days
						*MDADI:* Immediate decline post‐op; return to baseline at final follow‐up
					LOS	4.2 ± 1.9 days (range 2.4–5.4 by site)
Ma et al., 2023 [19]	Phase Ib/II single‐arm clinical trial	13		Immunotherapy (3 patients received durvalumab alone; 16 received durvalumab + tremelimumab) SBRT + TORS	Tumor response	
					Locoregional recurrence	5/19 patients (26%). In‐field: 1 patient Marginal: 1 patient Regional nodes: 3 patients
					Distant metastasis	None
					Median interval from last immunotherapy to recurrence	3 months
					Association with pathologic complete response	Failure to achieve pathologic complete response was significantly associated with locoregional recurrence (*p* = 0.033)
					Association with clinical T stage	No significant difference (*p* = 0.034)
					PD‐L1 expression	No significant differences in PD‐L1 tumor proportion score or combined positive score between patients with or without pathologic complete response, or between those with or without recurrence.
					PFS	*1‐year PFS:* 79% (95% CI: 52%–91%).
Nichols et al., 2019 [10]	Phase II randomized multicenter open‐label clinical trial (ORATOR)	68	Oropharyngeal	RT (70 Gy/35 fractions + chemo for N+) vs. TORS + ND	QOL – MDADI (1‐year)	*RT group:* 86.9 (SD 11.4); TORS Group: 80.1 (SD 13.0); *p* = 0.042
					FOIS (normal diet)	*RT group:* 100%; *TORS group:* 84%; *p* = 0.055
					Grade ≥ 2 toxicity	*RT group:* 91%; *TORS group:* 97%; *p* = NS
					G‐tube at 1 year	*RT group:* 3%; *TORS group:* 0%; *p* = NS
					3‐year OS	*RT group:* 89%; *TORS group:* 87%; *p* = 0.82
					Toxicity profile	*RT group:* more hearing loss, neutropenia, tinnitus; *TORS group:* more trismus, oral bleeding (1 fatal hemorrhage)
Nichols et al., 2022 [16]	Long‐term results of the ORATOR Trial (median 45 months)	68	Oropharyngeal	RT (70 Gy/35 fractions + chemo for N+) vs. TORS + ND	QOL – MDADI (median 45 months)	Similar in both groups, no significant difference
					FOIS (normal diet)	*RT group:* 100% *TORS group:* 84% (*p* = 0.055)
					Grade ≥ 2 toxicity	*RT group:* 91%; *TORS group:* 97%; *p* = NS
					G‐tube at 1 year	*RT group:* 3%; *TORS group:* 0%
					OS	*RT group:* 87.2%; *TORS group:* 88.2%; p16+ → OS and PFS significantly better vs. p16− (*p* < 0.001)
					PFS	*RT group:* 87.3%; *TORS group:* 85.3%; p16+ → OS and PFS significantly better vs. p16− (*p* < 0.001)
					Toxicity profile	*RT group:* more hearing loss, neutropenia, constipation, tinnitus; *TORS group:* more trismus, weakness, pain
O'Hara et al., 2024 [11]	Phase III randomized clinical trial (substudy of PATHOS)	508	Oropharyngeal	TLM vs. TORS	LOS	TORS associated with *longer hospitalization* (median: 5 days vs. 3 days for TLM; HR = 0.66, 95% CI: 0.55–0.79). Difference *not significant* after adjusting for treating center (HR = 0.89, 95% CI: 0.69–1.16).
					Use and duration of NGT feeding	*Higher rate* with TORS (45.0% vs. 7.9% for TLM; OR = 9.48, 95% CI: 4.68–19.22). Remained significant after adjustment (OR = 4.41, 95% CI: 1.01–19.32). NGT duration: *No significant* difference (median: 5 days for TLM vs. 6 days for TORS).
					Swallowing function (water swallow test, VFSS)	*Water swallow test:* No clinically meaningful difference. VFSS: *Aspiration:* higher rate with TORS (13.4% vs. 4.7% for TLM; OR = 3.29, 95% CI: 0.95–11.40). *High‐grade dysphagia:* more frequent with TORS (18.3% vs. 8.2%; OR = 3.32, 95% CI: 0.81–13.56).
					QOL	*H&N35 (swallowing):* worse with TORS (+7.24 points, 95% CI: 2.17–12.30).
					Postoperative hemorrhage	Low and similar rates (TLM: 1.0% vs. TORS: 1.6%).
Palma et al., 2025 [12]	Phase II randomized open‐label clinical trial (ORATOR2)	61	Oropharyngeal (HPV positive)	TLM/TORS + ND vs. RT	OS	*RT arm:* 2‐year OS was 100% (95% CI: 100%–100%); TLM/TORS + ND arm: 90% (95% CI: 71%–97%) (log‐rank *p* = 0.041).
					PFS	*RT arm:* 2‐year PFS was 100% (95% CI: 100%–100%); TLM/TORS + ND arm: 86% (95% CI: 67%–95%) (log‐rank *p* = 0.012)
					Recurrences	All three in the TLM/TORS + ND arm
					Toxicity (CTC‐AE)	*RT arm:* Grades 2–5 toxicities occurred in 70%; TLM/TORS + ND arm: 77% (*p* = 0.51); two deaths in the surgical arm due to transoral bleeding and cervical osteomyelitis.
					QOL	*MDADI:* mean 2‐year MDADI total scores were 89 ± 13 (RT) vs. 83 ± 11 (TOS + ND) (*p *= 0.11). The RT arm had significantly better scores in the physical subdomain over time (*p *= 0.02).
						*EORTC H&N35:* The RT arm had significantly better scores in social eating, coughing, and painkiller use. The TOS + ND arm had better scores in the sensory subdomain (taste + smell).
Park et al., 2017 [17]	Single‐arm clinical trial	31	Tonsil and tongue base	Neoadjuvant CTx + TORS + customized adjuvant therapy (surgery only or RT/CCRT) – 27/31 patients (87.1%) with adverse pathologic features received RTx or CCRTx per institutional protocol; 4 patients (12.9%) with no adverse features received no adjuvant treatment.	Local recurrence	0 cases
					Regional recurrence	Four patients (12.9%)
					Distant metastasis	One patient (3.2%)
					5‐year OS	78.7%
					5‐year DSS	85%
					5‐year DFS	80.8%
					5‐year OS	90% (p16+) vs. 70% (p16−)
					5‐year OS (p16+ vs. p16‐)	90% vs. 70%
					Reconstruction	Three patients with Alloderm; others healed by secondary intention
					Airway management	*Fifteen patients* → elective tracheotomy (decannulation mean: 7.1 days). *Sixteen patients* → nasotracheal intubation, extubated immediately after surgery
					Feeding	All patients resumed oral diet at a mean of 7.4 days postoperatively
					Swallowing (FOSS scale)	83.9% (Scores 0–2) → favorable outcome. 16.1% (Scores 3–4) → unfavorable outcome. No patient remained feeding tube–dependent.
					Breathing/phonation	All patients breathing and speaking without permanent tracheostomy.
					Nasometry	No hypernasality; all scores within normal ranges
Rubek et al., 2017 [13]	Phase II single‐arm clinical trial	30	Oropharyngeal	TORS + ND	Adjuvant therapy	Thirty patients (43%) were eligible for postoperative adjuvant therapy
					Margin status	Radical resection with free margins (≥ 2 mm) in 29/30 patients
					Stage migration	Twelve patients were upstaged at the N‐site. Two patients were upstaged from cT1 to pT2.
					Complications	No intraoperative complications. One patient had severe postoperative bleeding requiring readmission and ICU care. Two patients had minor postoperative bleeding not requiring intervention. Three patients had nosocomial suspected aspiration pneumonia.
					NGT	4.6 days (range 0–19 days)
					Los	5.3 days (range 2–13 days)
					Long‐term side effects	One patient developed persistent hypernasal speech.
Swisher‐McClure et al., 2019 [14]	Phase II single‐arm clinical trial (NCT02159703)	60	Oropharyngeal	ND + TORS + DART targeting the at‐risk lymph node	Local control	2‐year local control rate was 98.3%. One patient (1.7%) developed a local recurrence.
					Regional control	2‐year regional recurrence‐free survival was 97.9%. One patient (1.7%) developed a regional failure.
					Distant control	2‐year distant metastasis‐free survival was 96.2%. Two patients (3.3%) had distant failure.
					OS	100%. Progression‐free survival was 92.1% at 2 years.
					Toxicity	No patients required a feeding tube during RT. Two patients (3.3%) required feeding tubes during follow‐up (both subsequently removed). Soft tissue necrosis within the TORS site in two patients (3.3%);
Theurer et al., 2024 [18]	Prospective cohort (substudy of ORATOR)	21	Oropharyngeal	RT vs. TORs	Dysphagia	DIGEST ≥ 2 (mild to moderate)
					Swallowing	*6 months:* swallowing impairments increased in both arms, with some patients showing aspiration (highest PAS = 8 in RT, 6 in TORS). ~20% had moderate‐to‐severe dysphagia. *12 months:* Partial recovery observed; most patients had none‐to‐mild dysphagia, but ~25% had moderate dysphagia. No severe dysphagia reported. No significant differences between RT and TORs arms in objective swallowing measures.

Abbreviations: CCRTx, concurrent chemoradiotherapy; CTC‐AE, common terminology criteria for adverse events; CTx, chemoradiotherapy; DART, deintensified adjuvant radiotherapy; DIGEST, Dynamic Imaging Grade of Swallowing Toxic Effects; EORTC QLQ‐C30, European Organization for Research and Treatment of Cancer Quality of Life Questionnaire‐Core 30 [28]; FOIS, Functional Oral Intake Scale; QLQ‐H&N35, disease‐specific EORTC QLQ module for HNC [29]; LOS, length of hospital stay; MDADI, MD Anderson Dysphagia Index; ND, neck dissection; NGT, nasogastric tube; OS, overall survival; PEG, percutaneous endoscopic gastrostomy; PFS, progression‐free survival; QOL, quality of life; RT, radiotherapy; SBRT, stereotactic body radiotherapy; TLM, transoral laser microsurgery; TORS, transoral robotic surgery; VFSS, videofluoroscopy.

According to the SR by Roselló et al. [20], the operative time is shorter in the TORS group (except in cases requiring mandibulectomy), and the blood loss is lower. Fistulas only occurred in the open surgery group and there was no difference in positive margins among treatment modalities. Functional outcomes differences were impressive, with the TORS group having a shorter length of hospital stay (LOS) (3.8–26.1 days vs. 8–43.4 days with open surgery) and a faster recovery regarding decannulation and oral feeding. Among the studies included in this SR, recurrence‐free survival (RFS) and overall survival (OS) were better in the TORS group, except for one study [30] that showed similar results. This finding may suggest that the improved results with TORS are partly due to the selection of patients with more favorable clinical characteristics and smaller tumors compared with those undergoing the open approach (Table 2).

**Table 2:** Comparison among transoral robotic surgery (TORS), transoral laser microsurgery (TLM), and conventional open surgery across key aspects.

Aspect	TORS	TLM	Open surgery
Visualization	3D HD, excellent access to deep areas	2D, good for superficial lesions	Limited, requires wide exposure
Margin control	High (deep margin control)	Moderate (visibility‐dependent)	Variable
Complications	Hemorrhage, trismus	Low morbidity, hemorrhage	Surgical access related: visible scars mandibulotomy complications: ORN, tooth loss, nonunion cutting through healthy tissues for access (named nerves, muscles, etc.) Resection‐related communication with open neck: fistulas, infection, hemorrhage, trismus
Hospital stay	Short (3–7 days)	Very short (2–5 days)	Long (8–14 days or more)
Tracheostomy need	Low	Very low	High (especially in laryngeal cases)
Feeding tube dependency	Moderate	Low	High (especially in extensive cases)
Accessibility	Limited (high cost, restricted availability)	High (widely available)	Universal
Ideal indications	HPV+ oropharyngeal, supraglottic T1–T3	Superficial laryngeal lesions T1–T2	Advanced tumors, poor exposure cases
Cost	High	Low	Variable
Learning curve	Moderate to high	High	High

Abbreviation: ORN, osteoradionecrosis.

As previously stated by Boehm et al. [21], there is a notable lack of prospective randomized clinical trials comparing TORS to standard treatments, hindering objective assessment. In our ScR, we have identified 13 clinical trials in which the included clinical trials comprised a heterogeneous set of study designs. Six articles reported randomized controlled trials [10, 11, 12, 15, 16, 18], providing the highest level of evidence through random allocation of participants. One study adopted nonrandomized comparative design [7]. In contrast, eight investigations [5, 6, 8, 9, 13, 14, 17, 19] were conducted as single‐arm trials, in which all patients received the same intervention without a control group. Four clinical trials compared TORS with conventional treatment modality as RT [10, 12, 16, 18]. These results highlight the methodological diversity across the available literature, with implications for the level of evidence.

#### 3.3.2 Comparison Between TORS and Transoral Laser Microsurgery (TLM)

Regarding the comparison with transoral laser microsurgery (TLM), TORS has the advantages of enhanced visualization (the 3D high‐definition imaging improves surgical precision and access to deep anatomical areas like the base of tongue and supraglottis), improved manipulation (robotic arms mimic wrist movements, allowing better dexterity and tremor filtration), more flexibility in accessing deep or complex anatomical regions, resulting in better deep margin control, and surgeon comfort. Otherwise, TORS has a higher cost (robotic systems are expensive and not universally available, the expensive instruments have a limited lifespan) and requires longer setup time. Long‐term speech and swallowing outcomes are generally excellent with both techniques, with low rates of permanent tracheostomy or feeding tube dependency. In the available studies, TLM was associated with lower short‐term morbidity, including reduced rates of feeding tube placement and tracheostomy during early recovery [11, 31, 32]; however, this finding may be confounded by case selection, as TLM is often preferentially used for less extensive disease. Both TORS and TLM are effective minimally invasive techniques for treating HNCs. TORS offers superior visualization and access, making it ideal for complex or deep‐seated tumors. TLM, on the other hand, remains a reliable and accessible option for early‐stage and superficial lesions, particularly in the larynx (Table 2).

#### 3.3.3 Applications

Robotic surgery enhances precision through 3D visualization, fine motor control, and articulated instruments, enabling operations in confined anatomical spaces.

The SR performed by Boehm et al. [21] concludes that TORS is routinely used for early‐stage (T1–T2) oropharyngeal tumors, showing good functional outcomes (swallowing, QOL) [33] and comparable survival rates to open surgery. A study by Park and colleagues also found no difference in 5‐year survival between TORS and open surgery, but noted that TORS offered a shorter recovery time, reduced hospitalization, and better swallowing function. However, the problem inherent in all these uncontrolled studies is, of course, selection bias, as more complex and advanced cases are typically selected for the open approach.

Selected malignant neoplasms of the larynx, especially supraglottic tumors (T1–T3), when there is adequate exposure, are feasibly approached with TORS with advantages in visualization, reduced need for tracheostomy, and better phonation as compared to open approaches (limited evidence) [34].

TORS also shows potential for skull base and paranasal sinuses as clivus chordomas and nasopharynx tumors via transpalatal access, but challenges include lack of specialized instruments (e.g., drills for bone removal), and complications (i.e., velopharyngeal insufficiency and fistulas). Concerning thyroid and neck dissection, esthetic benefits are highlighted; transaxillary/retroauricular/transoral robotic approaches reduce visible scarring, but oncologic safety remains a priority. In addition, TORS enables facilitated access to retropharyngeal lymph nodes, enabling histologic confirmation of metastases and avoiding unnecessary adjuvant therapy [23].

#### 3.3.4 Technological Developments

The literature review by Poon et al. [24] analyzed the evolution, adoption, and clinical outcomes of robotic systems for TORS. The foundations of robotic surgery trace back to NASA's 1950s vision of telemedicine, providing remote surgical care to astronauts in space. This concept inspired Stanford Research Institute (SRI) to develop battlefield telesurgery in the 1990s, aiming to treat wounded soldiers remotely. Intuitive Surgical's Da Vinci System (derived from SRI tech) was developed and commercialized in early 2000 with FDA approval for TORS in 2009 (benign/T1–T2 malignancies). This system technical breakthroughs encompassed multiarm design allowing bimanual operation, 5 mm EndoWrist instruments (for the Da Vinci S and Si systems) with 7 degrees of freedom and surgeon console with 3DHD vision (10–12× magnification). The clinical impact was in the order of less blood loss, shorter hospital stay, and less tracheostomy dependence. However, limitations persist, and for TORS these have even increased with the latest multiport system, the Da Vinci Xi generation, due to its large instrument profile (8 mm shafts) and restricted access in patients with limited mouth opening (< 3cm), retrognathia and anterior commissure involvement [24]. The Da Vinci Single‐Port (SP) Robotic System, originally developed for urology, has shown significant benefits for TORS, as it allows flexible control of the instruments and camera, improving access to narrow spaces. Its design allows three robotic arms to be inserted through a 2.5 cm single port, while the endoscope arm can be adjusted into a “cobra head” configuration for enhanced exposure, particularly useful in hypopharyngeal and laryngeal surgeries. Advantages include shorter docking time, reduced instrument conflicts, improved maneuverability, faster learning curve, and shorter operative times. However, current tools are still often oversized for head and neck anatomy [21, 23].

The era of flexible robotics emerged to overcome the limitations of the Da Vinci system. Flexible instruments are particularly essential for TORS of the larynx, a need that was initially addressed by the prototype of a video laryngoscope with flexible instruments proposed by Schild et al. [35]. To surgically access the nasopharynx or the skull base with the Da Vinci system, a proposal was done to perform an invasive procedure called palate splitting (division of the hard and/or soft palate) [21]. This approach may lead to complications such as velopharyngeal insufficiency (difficulty in closing the palate during speech and swallowing) or fistula formation. Among the potential solutions, the Flex System (Medrobotics, Raynham, USA) has been designed as a flexible robotic platform capable of reaching anatomically challenging regions, while continuum robots, with their articulated and tubular structure, offer the ability to navigate through curved anatomical pathways with high precision [21, 36, 37]. Key innovations encompassed snake‐like articulated arm, single‐port access, and integrated accessory channels [24]. The Flex Robotic System aimed to be a significant advancement in minimally invasive head and neck surgery, with primary applications in the oropharynx, hypopharynx, and laryngeal regions (including supraglottis and glottis). This system claimed to offer several distinct advantages: its specialized design for head and neck anatomy would provide excellent adaptation to narrow pharyngeal spaces, eliminate the need for head reclination, and would enable surgical access even in patients with limited mouth opening. However, the technology was hampered by significant limitations, including a time‐consuming and technically demanding positioning process that frequently requires manual adjustments. Ultimately, these challenges, combined with commercial failure, led to the company's collapse in 2021 and the discontinuation of the Flex system. Consequently, the platform failed to meet clinical expectations and is now regarded as a promising but ultimately unsuccessful technology that did not deliver on its initial potential.

Haptic feedback and smaller instruments are needed for laryngeal microsurgery. The Versius and Senhance systems represent innovative haptic feedback robotic platforms, initially developed for abdominal surgery but demonstrating promising potential in head and neck applications. These systems offer three key advantages: flexible robotic ports that enhance surgical access, haptic feedback providing critical tactile sensation for improved safety, and reusable instruments that significantly reduce procedural costs. However, several limitations currently restrict their adoption in head and neck surgery: the Versius holds only CE certification, while both systems lack FDA/CE approval specifically for head and neck procedures. Clinical implementation remains limited to select hospitals, compounded by the absence of specialized instruments designed for head and neck anatomy and inadequate adaptation to the confined surgical fields characteristic of this anatomical region. These factors currently position haptic systems as emerging technologies requiring further development before becoming mainstream options in head and neck surgical oncology [21]. It is good to know that in Liverpool a single‐arm prospective safety and feasibility study using the Versius system in head and neck surgery started in December 2023 and completed inclusion of 60 patients in April 2025; results are pending but will be available in the coming months (NCT06112535).

#### 3.3.5 Current Limitations

Limited instrumentation and the lack of robust prospective randomized trials remain significant barriers. Existing cost data are primarily derived from small, nonrandomized, or uncontrolled studies. Few trials provide direct comparisons of TORS with established approaches, such as transoral laser surgery or open techniques. Early evidence suggests TORS is feasible and may offer benefits, like reduced tracheostomy dependence, but current studies are constrained by small sample sizes, absence of control groups, or heterogeneous comparators. While high‐quality randomized controlled trials are needed to confirm its efficacy relative to standard treatments, several major trials have recently completed accrual and are awaiting data maturation and analysis. These include:

- The EORTC 1420 “Best of” trial (NCT02984410), which randomized 86 patients with early‐stage oropharyngeal, supraglottic, and hypopharyngeal cancer to radiotherapy or transoral surgery (TORS/TLM) with or without adjuvant therapy [38].
- The DAHANCA QOLATI trial (NCT04124198), which has met its enrollment goal of 138 patients with early‐stage oropharyngeal squamous cell carcinoma (OPSCC) randomized to TORS versus intensity‐modulated radiotherapy.
- The EORTC 1701 “PATHOS” trial, a large‐scale study that accrued 1269 patients to evaluate de‐intensified adjuvant therapy based on risk factors following TORS [39].

Future research should focus on functional outcomes, cost‐effectiveness, and the development of standardized surgical protocols [20, 21, 22, 23, 24].

#### 3.3.6 Emerging Technologies and AI Integration

AI has been evolving in surgery since the first computer‐assisted systems in the 1980s, providing advances in personalized diagnosis, preoperative planning, intraoperative assistance, and postsurgical care. Its ability to continuously learn from large volumes of data promises greater precision, safety, and better outcomes. However, AI cannot yet replace manual surgery, as it cannot handle unpredictable situations. Thus, the role of the physician remains central, uniting technical skills, clinical judgment, and human values such as empathy and the doctor–patient relationship. The future of surgery, therefore, must balance technological innovation and fundamental surgical principles, ensuring standardization, safety, and preserving the humanistic essence of medical practice [25].

According to Kok Wah et al. [26], future research in AI‐driven robotic surgery should prioritize large, multicenter randomized controlled trials to validate efficacy across different populations and tumor types. Efforts must focus on developing explainable AI algorithms to build surgeon trust and ensure accountability, as well as integrating multimodal imaging, such as tactile sensing, 3D ultrasound, and augmented reality, to enhance intraoperative guidance in complex tumor locations. Longitudinal studies are also needed to assess long‐term oncologic outcomes, recurrence, and patient‐reported QOL, moving beyond feasibility and short‐term technical success. Additionally, cross‐disciplinary investigations into healthcare economics, policy, and ethics will be crucial for sustainable and equitable implementation. Overall, while early evidence highlights significant potential in precision and personalization, these innovations remain preliminary and require robust validation before widespread clinical adoption.

Only one study specifically addressed AI‐driven robotic surgery in the head and neck. Zhao et al. [27] investigated the use of electromagnetic navigation combined with intraoral robotic osteotomy for the management of mandibular tumors. Their preclinical, model‐based study demonstrated enhanced accuracy in jaw resections, suggesting the potential of this technology to improve precision in surgical procedures. Despite its transformative potential in head and neck oncology, AI adoption remains constrained by clinician mistrust, data heterogeneity, algorithmic bias, and ethical concerns. Overcoming these challenges through transparency, robust validation, and clinician engagement is critical for safe and equitable clinical integration [40, 41].

## 4 Discussion

In recent years, similar SRs on the topic have primarily aimed to compare TORS with nonrobotic surgery for OPSCC [42, 43, 44], assess its applications [45] and indications [46], or evaluate functional outcomes [47] and QOL [48]. Our ScR differentiates itself and justifies its contribution by adopting a distinct and comprehensive purpose and offering a panoramic and forward‐looking overview of the state of the art and the trajectory of TORS. Our primary objective was to map the advances in robotic surgery for head and neck oncology, explore the latest technological developments, discuss current limitations, and present ongoing projects and future perspectives. To fulfill this aim, we prioritized robust evidence: clinical trials were assessed to retrieve the most current and high‐quality evidence on TORS, while existing reviews and meta‐analyses were evaluated to synthesize recent advances, emerging technologies, existing challenges, and future directions in the field.

Among studies that compared TORS + ND with RT [10, 12, 16], all three evaluated patients with oropharyngeal cancer and focused on safety and efficacy outcomes. According to the ORATOR Trial [10, 16], the 3‐year OS was similar between the groups, whereas in the ORATOR2 trial [12] the 2‐year OS and PFS were better in the group that received RT (log‐rank *p *= 0.041). Regarding toxicity and adverse events, in the ORATOR2 trial [12], Grades 2–5 toxicity was similar between the groups. In contrast, the ORATOR Trial [10, 16] found that the TORS group had more hemorrhage and trismus, while the RT group had more hearing loss, neutropenia, and tinnitus, findings that were later confirmed by the same research group [16]. However, the generalizability of these findings from the ORATOR trials has been met with significant criticism from the surgical community. Key methodological concerns include the inclusion of a high proportion of patients with N2 disease (approximately 50%), which necessitated multimodality therapy in most surgical patients, thereby confounding the assessment of TORS as a primary treatment. Furthermore, the protocol mandated universal tracheostomy for TORS patients, a practice not representative of contemporary standard care, which likely inflated the perceived morbidity of the surgical approach. These factors suggest that the trials' conclusions may reflect an outdated surgical paradigm rather than the current safety and efficacy profile of TORS in appropriately selected patients [49]. Concerning QOL, while the ORATOR2 trial [12] showed no significant differences, the ORATOR Trial [10] reported a more favorable QOL for the RT group (*p *= 0.042). Later, Nichols et al. [16] reported that QOL was similar in both groups after a median follow‐up of 45 months. A critical distinction lies in the toxicity profiles of each modality. The surgical approach carries a characteristic risk of procedure‐specific adverse events, such as postoperative hemorrhage and trismus. In contrast, RT is associated with a different set of toxicities, often involving systemic effects (e.g., neutropenia) and otologic sequelae (e.g., hearing loss, tinnitus). This dichotomy highlights the importance of tailoring treatment selection to individual patient risk factors and priorities. In contrast, TORS followed by reduced‐dose postoperative radiotherapy (PORT) (50 Gy) in intermediate‐risk HPV‐positive oropharyngeal cancer achieved excellent progression‐free survival (PFS) and OS, with lower toxicity and better QOL compared to standard‐dose (60 Gy) or intensified (66 Gy plus cisplatin) adjuvant therapy. Further underscoring the complexity of functional outcomes, Theurer et al. [18] compared RT with TORS for early OPSCC and reported that both are associated with swallowing impairment post‐treatment, but functional outcomes appear comparable between arms at 12 months. Despite recovery, a subset of patients (~25%) continue to experience clinically meaningful dysphagia 1 year after treatment. Functional swallowing impairment correlates only weakly with patient‐reported QOL, highlighting the need for multidimensional assessment.

The authors, representing the German Society of Otorhinolaryngology, Head and Neck Surgery (Oncology Working Group), caution against drawing conclusions for clinical practice. They emphasize the main limitations of the ORATOR2 trial, particularly in the surgical arm, including the requirement of at least 1 cm margins (with re‐operation recommended if not achieved), the prohibition of regional or free flaps, high tracheotomy rates, and a low proportion of TLM. In addition, the small sample size, highly selected patient cohort, and short follow‐up period further restrict the study's applicability. Consequently, patients with HPV‐related OPSCC should not receive de‐escalated (radiation) therapy outside of clinical trials. Until the results of ongoing trials (e.g., EORTC 1420) are available, treatment decisions between surgical and radiotherapeutic approaches should be made through interdisciplinary tumor board discussions, ensuring patients are fully informed about the benefits and drawbacks of each modality [50].

In single‐arm clinical trials investigating TORS, Dabas et al. [6] and Holsinger et al. [9] evaluated patients with oropharyngeal cancer, while Chan et al. [5] included a wider variety of anatomical sites in addition to the oropharynx. All studies reported low conversion rates and relatively small blood loss. However, there was considerable variation in the LOS, with Chan and colleagues reporting a mean LOS of 10 days, compared to 5.8 days in Dabas and colleagues and 4.2 days in Holsinger and colleagues. Chan's longer LOS is possibly due to differences in the patient population or institutional protocols. The estimated blood loss also varied drastically. Regarding complications and adverse events, all studies demonstrated that severe complications are rare, with the majority being manageable.

Among clinical trials investigating TORS with ND as a primary treatment modality, several key consistencies and distinctions emerge. The studies consistently focused on patients with oropharyngeal cancer, unanimously reporting TORS to be a viable and safe technique. Intraoperative complications were rare to absent, and most postoperative complications, such as minor hemorrhage or pneumonia, were manageable. All trials demonstrated excellent local and regional disease control, evidenced by high rates of radically resected free margins [13] and impressive 2‐year local and regional control rates [14]. However, the studies differed in their specific focus and scope. Haller et al. [8] provided data from a substantially larger cohort (*n* = 219 vs. *n* = 30), offering greater statistical power for quantifying complication rates, such as a precise postoperative bleeding rate of 13.2% and a 4.6% rate of operative re‐intervention. In contrast, Rubek et al. [13] offered a more detailed analysis of specific functional and perioperative outcomes, including the use of nasogastric tubes (NGT) and tracheostomy. A fundamental difference lies in the research objective of the Swisher‐McClure et al. [14] trial. This study utilized TORS as a strategic tool to enable treatment de‐escalation, specifically by reducing the intensity of adjuvant RT. Consequently, its primary endpoints were long‐term oncologic efficacy (survival, disease control) and late RT‐related toxicity, rather than surgical complications. In conclusion, this body of evidence confirms that TORS is a secure technique with two primary applications: it can serve as a definitive treatment (followed by adjuvant therapy as indicated by pathology) or as an initial step (“de‐intensification strategy”) to facilitate less toxic combined‐modality treatment regimens.

This ScR clearly delineates critical gaps and priorities for future research. The most pressing need remains the generation of high‐level comparative evidence. While trials like PATHOS, QOLATI, and the “Best of” trial have completed accrual, their mature data on oncologic and functional outcomes are eagerly awaited. Future efforts must build on this by initiating large, multicenter randomized controlled trials that directly compare TORS not only to open surgery but, more critically, to other organ‐preservation strategies like TLM and radiotherapy, with a primary focus on patient‐reported QOL and functional outcomes. Furthermore, this review highlights an urgent need for robust health economic analyses. Current cost data are derived from heterogeneous, often nonrandomized studies. Future research must employ standardized methodological designs to perform rigorous cost‐effectiveness analyses, determining the true value proposition of TORS for healthcare systems. Finally, to overcome the technical barriers identified, a concerted translational effort is essential. Future engineering and clinical research should prioritize the development and validation of next‐generation technologies, including smaller, specialized instruments for confined spaces, haptic feedback systems, and explainable AI platforms for surgical planning and navigation, to safely expand the applications of robotic surgery in the head and neck.

A primary constraint of this ScR is the pronounced methodological and clinical heterogeneity of the included evidence. The literature comprises a mix of randomized trials, nonrandomized comparative studies, and single‐arm cohorts, with significant variations in tumor sites, stages, and treatment protocols. This diversity precluded a quantitative synthesis and challenges the formulation of definitive, unified conclusions. Furthermore, the body of evidence is characterized by a preponderance of small‐scale studies and early‐phase clinical trials, many of which are inherently limited by their sample size, lack of control groups, or short follow‐up, increasing the risk of overestimating efficacy and underreporting long‐term adverse events. Second, the rapid pace of technological innovation in robotic surgery presents a challenge for any systematic assessment. This review, based on the published literature, may not encompass the most recent advancements in single‐port systems, flexible robotics, or AI integration, whose clinical data are still emerging. Finally, as with any review, there is a potential for publication bias, where studies with positive results are more likely to be published, potentially skewing the overall perception of TORS’ benefits. The nascent field of AI in surgery is further constrained by fundamental challenges, including data heterogeneity, algorithmic bias, and the “black box” problem, which currently limit its reliable clinical application and are reflected in the scarcity of high‐level evidence in this review [40].

## 5 Conclusions

While TORS has become a well‐established and valid technique for selected HNCs, offering a minimally invasive alternative on par with TLM, it has not yet demonstrated definitive superiority over traditional open surgery. For TORS to progress from a promising option to an unequivocal standard of care, future efforts must focus on three critical areas: (1) generating Level I evidence through mature, large‐scale randomized controlled trials to prove its comparative effectiveness; (2) overcoming major barriers related to high costs and instrument limitations through technological innovation and cost‐reduction strategies; and (3) rigorously validating the integration of advanced technologies like AI to enhance surgical precision, safety, and patient outcomes. The next decade of TORS will thus be defined not by its technical feasibility, but by its proven value and continued technological refinement.

## Author Contributions

All authors have made substantial contributions to the conception (Anna Luíza Damaceno Araújo, Alfio Ferlito, Luiz Paulo Kowalski), draft and design (Anna Luíza Damaceno Araújo, Karthik N. Rao, Alfio Ferlito, Luiz Paulo Kowalski), data acquisition (Anna Luíza Damaceno Araújo, Alfio Ferlito, Luiz Paulo Kowalski), manuscript preparation (Anna Luíza Damaceno Araújo), editing (Anna Luíza Damaceno Araújo, Karthik N. Rao, Juan P. Rodrigo, Vincent Vander Poorten, Orlando Guntinas‐Lichius, Alfio Ferlito, Luiz Paulo Kowalski), and review (Anna Luíza Damaceno Araújo, Karthik N. Rao, Juan P. Rodrigo, Vincent Vander Poorten, Orlando Guntinas‐Lichius, Alfio Ferlito, Luiz Paulo Kowalski) of the paper's final version. The authors agree to be accountable for all aspects of the work and ensure that questions related to the accuracy or integrity of any part of the work are appropriately investigated and resolved. All authors made substantial contributions to the conception, draft and review of the data for the work.

## Funding

The authors have nothing to report.

## Ethics Statement

The authors have nothing to report.

## Conflicts of Interest

The authors declare no conflicts of interest.

## Synopsis

This scoping review maps the contemporary landscape of transoral robotic surgery (TORS) for head and neck cancer, synthesizing evidence that establishes it as a safe and effective minimally invasive technique. We find that while TORS offers a distinct morbidity profile compared to radiotherapy, it has not yet demonstrated definitive superiority, and its broader adoption is constrained by a need for higher‐level evidence and technological limitations. The future trajectory of TORS depends on generating robust comparative data through randomized trials and integrating next‐generation technologies like artificial intelligence and advanced instrumentation.

## Supporting information


Supporting File

## Data Availability

The data that support the findings of this study are available from the corresponding author upon reasonable request.
