# Supplementary material for: Transoral Robotic Surgery in Head and Neck Oncology: A Scoping Review of Evidence, Technological Advancements, and Future Trajectories
Source: J Surg Oncol. 2026 Aug 16;134(6):775–92. doi: 10.1002/jso.70353 (PMC13732544; doi:10.1002/jso.70353)
Supplement: Supplementary file 1 [file JSO-134-775-s001.docx]

**Supplementary Appendix 1.** Search strategy

| **Sources** | | **Strategy** | **Search results** | **Total** |
| --- | --- | --- | --- | --- |
| **Database** | Embase | ('head and neck cancer'/exp OR 'head and neck cancer') AND 'robotic surgery':ti,ab,kw | 1442 | **5282** |
|  | Pubmed/MEDLINE | "head and neck cancer" OR "oncology" and "robotic surgery" | 2802 |  |
|  | Scopus | ( TITLE-ABS-KEY ( "head and neck cancer" ) AND TITLE-ABS-KEY ( "robotic surgery" ) ) | 477 |  |
|  | Portal Regional da Biblioteca Virtual em Saúde* | "head and neck cancer" OR "câncer de cabeça e pescoço" OR "cáncer de cabeza y cuello" and "robotic surgery" OR "cirurgia robótica" OR "cirugía robótica" | 119 |  |
|  | Web of Science | “head and neck cancer” (All Fields) AND “robotic surgery” (All Fields) https://www.webofscience.com/wos/woscc/summary/6f5c33cb-e638-42fb-9c9f-ed50a92fd0ff-0173d5a150/relevance/1 | 442 |  |
| **Grey literature** | Google Scholar | “head and neck cancer” AND “robotic surgery” | 100 | **129** |
|  | ProQuest | title("head and neck cancer") AND title("robotic surgery") | 29 |  |
| **Total** |  |  | **5411** | |

*The Regional Portal of the Virtual Health Library includes the following databases: Base de Dados de Enfermagem (BDENF); Base de Dados de Odontologia (BBO); Base de Dados de Enfermagem da América Latina e Caribe (BDNPAR); Biblioteca Regional de Medicina – Literatura Latino-Americana e do Caribe em Ciências da Saúde (LILACS); Biblioteca Virtual de Saúde – Enfermagem (CUMED); BRISA/RedTESA (BRISA/RedTESA); Índice Bibliográfico Nacional em Ciências da Saúde (BINACIS); MEDLINE (MEDLINE); Ministério da Saúde do Chile (MINSALCHILE); Secretaria de Estado da Saúde de São Paulo (Sec. Est. Saúde SP)

**Supplementary Appendix 2:** Excluded articles and reasons for exclusion.

|  | **N** | **Author** | **Title** | **Year** | **Country** | **Journal/Conference name** | **Exclusion Criteria** |
| --- | --- | --- | --- | --- | --- | --- | --- |
|  | 1 | De Almeida et al. | Finding/identifying primaries with neck disease (FIND) clinical trial protocol: a study integrating transoral robotic  surgery, histopathological localization and tailored deintensification of radiotherapy for unknown primary and  small oropharyngeal head and neck squamous cell carcinoma | 2029 | USA | BMJ Open | Study protocol/preliminary results of clinical trial |
|  | 2 | Evans et al. | PATHOS: A phase II/III trial of risk-stratified, reduced intensity adjuvant treatment in patients undergoing transoral surgery for human papillomavirus (HPV)-positive oropharyngeal cancer | 2018 | UK | J. Clin. Oncol. | Conference Abstract |
|  | 3 | Nichols et al. | Early-stage squamous cell carcinoma of the  oropharynx: Radiotherapy vs. Trans-Oral Robotic  Surgery (ORATOR) – study protocol for a  randomized phase II trial | 2013 | UK | MC Cancer | Study protocol/preliminary results of clinical trial |
|  | 4 | Park et al. | The First Human Trial of Transoral Robotic Surgery Using a Single-Port Robotic System in the Treatment of Laryngo-  Pharyngeal Cancer | 2019 | Korea | Ann Surg Oncol | Study design (not clinical trial – retrospective design) |
|  | 5 | Rosenberg et al. | Randomized phase I/II trial of TheraT vectors expressing HPV16 specific antigens with neoadjuvant chemotherapy followed by transoral robotic surgery (TORS) or risk/response stratified chemoradiotherapy (CRT) for locoregional HPV16+ oropharyngeal cancer (OPC) | 2023 | USA | J. Clin. Oncol. | Conference Abstract |
|  | 6 | Sadeghi et al. | Neoadjuvant Chemotherapy and Transoral Robotic Surgery  for Human Papillomavirus–Related Oropharyngeal Cancer | 2024 | EUA | JAMA Otolaryngology–Head & Neck Surgery | Study design (not clinical trial – retrospective design) |
|  | 7 | Yan et al. | Neoadjuvant sintilimab and platinum-doublet chemotherapy followed by transoral robotic surgery for HPV-associated resectable oropharyngeal cancer: Single-arm, phase II trial | 2024 | China | J. Clin. Oncol. | Conference Abstract |
|  | 8 | Clinical Trial NCT04277858 | Phase II Study: Induction Chemotherapy Followed by Transoral Robotic Surgery and Neck Dissection for Definitive Management of Oropharyngeal Squamous Cell Carcinoma. (NECTORS Trial) | - | - | - | Clinical trial in progress |
|  | 9 | Clinical Trial NCT05387915 | Biomarker-Driven Radiation Dose Reduction After TORS in Patients With HPV-Positive Oropharyngeal Cancer | - | - | - | Clinical trial in progress |
|  | 10 | Clinical Trial NCT01953952 | Randomized Phase II Trial of Transoral Endoscopic Head and Neck Surgery Followed by Risk-Based IMRT and Weekly Cisplatin Versus IMRT and Weekly Cisplatin for HPV Negative Oropharynx Cancer | - | - | - | Clinical trial in progress |
